# Hydrogel Based on Nanoclay and Gelatin Methacrylate Polymeric Matrix as a Potential Osteogenic Application

**DOI:** 10.3390/jfb14020074

**Published:** 2023-01-29

**Authors:** Danielle B. Andrade, Leticya L. S. Soares, Francisca L. A. Cardoso, Idglan S. Lima, Jhaemely G. V. Silva, Maria A. M. Carvalho, Maria G. Fonseca, Guilherme de C. Brito, Francisco Eroni P. Santos, Josy A. Osajima, Anderson O. Lobo, Edson C. Silva-Filho

**Affiliations:** 1 Interdisciplinary Laboratory for Advanced Materials (LIMAV), Materials Science and Engineering Graduate Program (PPGCM), Technology Center, Federal University of Piauí (UFPI), Teresina 64049-550, PI, Brazil; 2 NUPCELT, Animal Science Center, Federal University of Piauí, Teresina 64049-550, PI, Brazil; 3 Research Center and Extension Laboratory of Fuels and Materials-NPE/LACOM, Department of Chemistry, Federal University of Paraíba, MGF, João Pessoa 58051-900, PB, Brazil; 4 Chemistry Department, Natural Science Center, Federal University of Piauí, Teresina 64049-550, PI, Brazil

**Keywords:** biomaterial, hydrogel, nanoclay, osteogenesis

## Abstract

A nanocomposite hydrogel has potentially applicability in the induction of osteogenesis. The hydrogel was synthesized using 1% gelatin methacrylate (GelMA), a biodegradable and bioactive polymer containing the structure of gelatin, denatured collagen derived from the extracellular bone matrix, and 6% laponite (Lap), a synthetic phyllosilicate of nanosized particles. Initially, 0.6 g of Lap was added to deionized water, and then a solution of GelMA/Igarcure was added under stirring and UV light for crosslinking. The spectra in the Fourier-transform infrared region showed bands that indicate the interaction between gelatin and methacrylate anhydride. X-ray diffraction patterns confirmed the presence of Lap and GelMA in the hydrogel. The thermogravimetric analysis suggested an increase in the thermal stability of the hydrogel with the presence of clay mineral. Rheological analysis showed that the hydrogel had a viscosity that allowed its injectability. The hydrogel did not show acute toxicity at any of the concentrations tested according to the *Artemia salina* lethality test. It showed cell viability more significant than 80% in the MTT test, which makes it suitable for in vivo osteogenic induction tests. The cell differentiation test showed the differentiation of stem cells into osteogenic cells. It indicates a material with the potential for osteogenic induction and possible application in bone tissue engineering.

## 1. Introduction

The search for bone tissue substitutes has been increasing in recent years. Tissue engineering is an interdisciplinary field that investigates three-dimensional structures formed from an artificial or natural material with high porosity and interconnectivity between pores, known as scaffolds, for tissue regeneration. Biocompatibility, absence of cytotoxicity and immunogenicity, biodegradability, and a three-dimensional structure suitable for the adhesion and survival of cells or growth factors are essential characteristics of scaffolds in tissue engineering [1,2,3].

Hydrogels are three-dimensional structures formed by polymers, synthetic or natural, which are cross-linkable and capable of absorbing a large amount of water [4]. Hydrogels mimic the natural extracellular matrix and control the release of active ingredients to tissues. They are biodegradable, and can be used as scaffolds, a basic structure that provides mechanical strength, a place for cell fixation, proliferation, and differentiation as well as for the transport and distribution of growth factors [5,6]. Hydrogels allow application through minimally invasive surgical procedures and are desirable as they reduce the risks associated with surgery, such as contamination [5,6,7]. Thus, injectable hydrogels are a viable alternative as they adapt to the surgical site and adhere to the surrounding tissues, enabling the treatment of areas that do not have a well-defined geometry [7].

Although hydrogels have important and promising properties for tissue engineering, due to their similarity to the cellular microenvironment, they still have some limitations, such as low mechanical stiffness, low stability, and accelerated degradation rate, which restrict their clinical application. Thus, it is necessary to induce modifications in the hydrogels to obtain better mechanical properties such as mechanical stability and/or elastic mechanical properties. Such improvements have been achieved with the introduction of groups that provide photocrosslinking. However, a study by Zhou et al., 2014 [8] showed that the hydrogel’s degree of crosslinking interferes with the mineralization process’s kinetics. The authors used gelatin methacrylate hydrogel (GelMA) as a template for hydroxyapatite mineralization. They concluded that hydrogels with a high crosslinking degree exhibited lower mineral content. Furthermore, the chemical composition of the crosslinker can affect the hydrogel diffusibility in the network. It becomes denser and more difficult for the mineralization precursor ions to be introduced; moreover, high levels of crosslinking are still cytotoxic, although this effect depends on the source of irradiation and the intensity of ultraviolet (UV) light. Therefore, the photoinitiator becomes biocompatible when photocrosslinking is performed under UV-light intensity and in a shorter exposure time [8,9].

GelMA is derived from gelatin, a natural polysaccharide, which has a variety of bioactive groups such as arginine–glycine–aspartic acid (RGD). It promotes cell adhesion and growth, matrix metalloproteinase (MMP), and endopeptidase, which support enzymatic degradation and are used for cell remodeling [10]. GelMa was obtained reacting the hydroxyl, amine groups of gelatin, and methacrylate anhydride in a phosphate-buffered solution at 50 °C in the presence of a photoinitiator. In addition, even with less than 5% methacrylate anhydride, GelMA has most of the functional amino acids of gelatin, including RGD and MMP. The RGD group does not have a functional group that can react with methacrylate anhydride, thus retaining the cell adhesion properties of gelatin in materials with GelMA. GelMA hydrogels are biocompatible, biodegradable, chemically reactive, and suitable for cell adhesion but have poor mechanical properties. The adjustment of the various parameters in the synthesis of GelMA makes it possible to regulate the mechanical and chemical properties of the resulting hydrogel. However, other properties such as degradability, pore size, and cell differentiation can be negatively affected [11]. 

Laponite (Lap), a nanoclay of the smectite group, is a lamellar silicate of magnesium and lithium, and has been the subject of much research. Due to its excellent potential for inducing osteogenic differentiation of human bone marrow mesenchymal cells (hBMSCs) even in the absence of of any other osteoinductive factor [12]. Clay nanoparticles are biocompatible and their degradation products are non-toxic, absorbable and relevant to osteogenic cell function, with good properties for use as biomaterial [13]. Lap has a particle size of 25–30 nm and 1 nm thickness, producing a surface area of 800 m^2^ g^−1^ and a cation exchange capacity of 80–150 meq/100 g. Anisotropy is a permanent negative charge on the surface and a positive on the edges, which gives it several possibilities for interactions with polymers or biomolecules [11,12,13,14,15].

As previously described, GelMA hydrogels have fragile mechanical properties that restrict their use in tissue engineering. Therefore, incorporating inorganic nanomaterials in these hydrogels has been an alternative route widely used to improve mechanical properties and may also increase biological ones [16]. The conjugation of a polymer with a nanomaterial allows the transfer of mechanical force within the reticulated networks, ensuring greater resistance to the hydrogel. These modified hydrogels also have advantages such as supporting viable cells, differentiating cells, and forming a stable vascular network [17,18].

Laponite is among the various options of inorganic nanomaterials used to associate with GelMA in synthesizing hydrogels. Studies also show that the combination of smectite clays with polymers in hydrogels can improve properties such as tensile strength, modulus of elasticity and compression, toughness, and viscoelasticity [13,14,15,16,17,18,19,20,21]. Laponite has also been used as nanocomposites with different polymers for biomaterials, highlighting its use with PEGDA. Different characterizations have demonstrated their potential and in vitro and in vivo tests for bone regeneration [22].

Here, in this work, a Lap/GelMA hydrogel was obtained to improve properties such as mechanical strength and biocompatibility to enable its application in bone tissue engineering. In addition, cytotoxicity assays indicate the biocompatibility of the gel produced, indicating a promising biomaterial.

## 2. Materials and Methods

### 2.1. Materials

The reagents were: Laponite (trademark of the company BYK Additives Ltda-Germany), deionized water (Milli Q^®^ water–Milli Q–Direct; Alphax, São Paulo, SP, Brasil), PBS (phosphate-buffered saline-Sigma–Aldrich, USA). The reagents used to prepare the saline water were: NaCl (Sodium Chloride P.A.-ACS—Dynamics–Brasil—99%); MgCl_2_ (Magnesium Chloride P.A.—Impex-Brasil—99%); MgSO_4_ (Magnesium Sulfate P.A.—Isofar—Brasil—99%); CaCl_2_ (Calcium Chloride P.A.—ACS—Dynamics–Brasil—99%); KCl (Potassium Chloride P.A.—ACS—Dynamics–Brasil—99%); NaHCO_3_ (Sodium Bicarbonate—Sigma—Aldrich, BioXtra—99.5–100.5%); Alizarin red (Sigma–Aldrich, USA); osteocyte/chondrocyte differentiation basal medium (Gibco Stem Pro, USA); and osteogenesis supplement (Gibco Stem Pro, USA).

### 2.2. Preparation of Hydrogel Lap/GelMA

Lap gel (trademark of the company BYK Additives Ltd.) at 6% was prepared by adding 0.6 g of Lap to a falcon tube containing 5 mL of Milli Q^®^ water at 4 °C. The solution was taken to a mechanical shaker for 15 min, at a rotation of 3000 rpm, until a clear gel was formed. The pH of the clay suspension was 10 without the use of further adjustment. [23]. The 1% GelMA solution was prepared using 0.1 g of GelMA and was added to a falcon tube containing 8 mL of saline solution—PBS (phosphate-buffered saline). The mixture was placed in an oven at 70 °C for 30 min [23,24]. After, 0.05 g of Igarcure 2959 (Ig) (Sigma) was placed in a beaker with aluminum foil containing 2.0 mL of PBS. The beaker was placed at 70 °C for 30 min under magnetic stirring at 1800 rpm. The photoinitiator solution was placed under mechanical agitation for 20 min at a rotation of 3000 rpm. Then, the Igarcure 2959 solution was transferred to the GelMA solution, already prepared. The mixture was placed on a magnetic stirrer at 40 °C with a rotation of 1800 rpm, for 15 min, protected from light.

The GelMA/Ig solution was added to a falcon tube containing the Lap gel. The tube was placed under mechanical agitation for 15 min, 3000 rpm, and the hydrogel was taken under a UV light source for crosslinking. The material was placed 10 cm from the light source of intensity 18.5 mW/cm^2^ for 2 min [23,24].

### 2.3. Characterizations

The material was previously lyophilized to carry out the characterizations. The short-range order was monitored by Fourier-transform infrared (FTIR) spectroscopy using a Perkin Elmer Spectrum 100, 4 cm^−1^ resolution, and 16 scans. X-ray diffractometry evaluated the long-range order obtained in a Shimadzu XDR—6000 diffractometer with a CuKα radiation source (λ = 1.5418 Å), 5–80° (2θ), 2 °C/min, operating at a voltage of 40 kV and 30 mA. Thermogravimetric analysis was performed with the samples in powder form, using SDT Q600-0883 (DSC-TGA) equipment from TA Instruments, with a heating rate of 10 °C min^−1^ and a flow rate of 100 mL min^−1^, under an argon atmosphere, using alumina crucible. All samples were measured with an AR-G2 rheometer (TA Instruments, New Castle, DE, USA) equipped with parallel-plate geometry. A Peltier base was used to control the 25 °C and 37 °C. A rheometer tested the storage modulus of the hydrogels in a time sweep procedure. Data analysis was performed with TRIOS software version 4.4.0.41651 (TA Instruments, New Castle, DE, USA). The material was lyophilized and prepared with and without metallization. The micrographs were performed in a scanning electron microscope (SEM) with a field emission gun, brand FEI, model Quanta FEG250, with acceleration voltage from 1 to 30 kV, equipped with EDS of SDD (silicon drift detectors), Ametek brand, model HX-1001, Apollo X-SDD detector.

### 2.4. Artemia Salina Assay

The *Artemia salina* lethality assay evaluated the toxicity of the hydrogel (*Artemia* eggs DE RP33801, JQ GmbH&Co.KG (Neuhofen, Germany). Initially, a saline water solution was prepared. A solution (10 mL) of the test material at 10,000 µg/mL concentration was produced and placed in a beaker. From this solution, 5 mL was removed and added to a test tube containing 5 mL of saline water, resulting in a concentration of 5000 µg/mL. From this solution, 5 mL was removed, and added to another test tube containing 5 mL of saline water, resulting in a concentration of 2500 µg/mL. The same procedure was carried out to obtain concentrations of 1250 µg/mL and 625 µg/mL, respectively. The experiment was performed in triplicate (10 nauplii in each tube). After 24 h, the number of still-alive nauplii was counted, and after 48 h, another count of live nauplii was made, and the LD 50 was calculated [25].

### 2.5. Cytotoxicity

**Figure 1:**
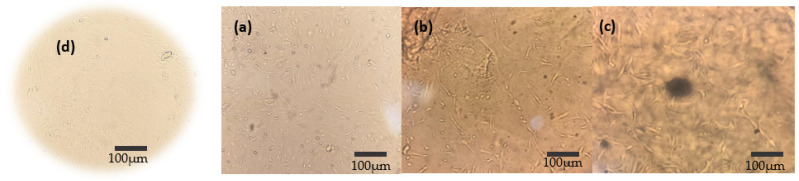
Images of cell viability tests by MTT: (**a**) GelMA 24 h, (**b**) Lap 24 h, (**c**) Lap/GelMA 24 h; (**d**) Control.

Cell viability was investigated by metabolic activity with the MTT assay (3-(4,5-dimethyithiazol-2-yl)-2,5-diphenyltetrazoliumbromid). Bone marrow stem cells from rats (Ethical Committee on Animal Experimentation—UFPI n°695/21) were cultured in Dulbecco’s Modified Eagles’s Medium (DMEM) containing 5% fetal bovine serum (Hyclone) and 1% (*v*/*v*) penicillin/streptomycin (Hyclone) in an incubator at 37°C and 5% CO_2_. Upon reaching 80% confluence, cells were removed and expanded through two passages until used. Cells were seeded in 96-well plates at a density of 1 × 10^5^ cells mL^−1^ and kept in culture for 24 h to form a semiconfluent layer (in triplicate). After this step, the cells were exposed to 0.0625 g of GelMA, laponite, and the Lap/GelMA hydrogel for 24 h, 48 h and 72 h, as seen in Figure 1 and Figure S1. Cells from the control wells were not exposed to the test sample. After the determined period, the culture medium was removed, and 50 µL of MTT solution was added and the plates were incubated for another 2 h at 37 °C—5% CO_2_. Then, the MTT solution was discarded and 100 µL of DMSO was added to the wells, and the plates were shaken and taken to the microplate reader to detect the absorbance at 570 nm. Cell viability was calculated by Equation (1) [26]:

Viab % = (100 × OD 570a)/OD570c (1)

where OD570a = average value of the optical density of the sample; OD570c = average value of the optical density of the control.

### 2.6. Osteogenic Differentiation Assssement

Osteogenic induction of bone marrow stem cells from rats in contact with the Lap/GelMA hydrogel and its precursors was performed by incubating the cells in 96-well plates at a density of 1 × 10^5^ cells mL^−1^ for each group in triplicate. After 70 to 80% confluence, all the medium was removed, and the osteogenic differentiation medium was added to the wells with laponite, GelMA, and hydrogel Lap/GelMA, respectively. Another group of wells containing cells and laponite, GelMA, and Lap/GelMA hydrogel was kept without an inducing medium. The other group of wells contained only cells with a culture medium (control). The medium was changed every 72 h, and after 21 days, fixation and staining were performed to observe cell differentiation. For staining, all the medium was removed, the wells were washed with PBS once and with distilled water, and then fixation was performed with 10% formalin, washed with distilled water, and Alizarin Red dye (2%) was added for 1 h.

### 2.7. Statistical Analysis

The statistical data were analyzed by analysis of variance (ANOVA) and the Tukey test using GraphPad Prism for Windows (GraphPad Software, San Diego, CA, USA). Data were expressed as mean ± SD. The control groups were compared with the other groups, with a statistically significant difference of *p* ≤ 0.05.

## 3. Results and Discussion

### 3.1. Structural Analysis of Hydrogel

**Figure 2:**
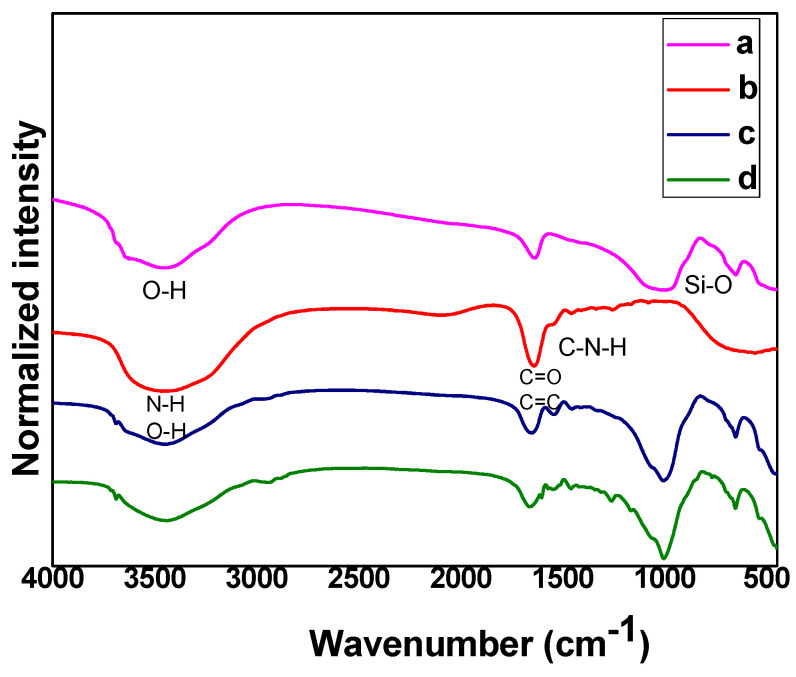
FTIR spectra (a) Lap; (b) GelMA; (c) Lap/GelMA hydrogel and (d) Lap/GelMA/IG (hydrogel with Igarcure 2959).

The FTIR spectra of the Lap/GelMA hydrogel and its precursors are shown in Figure 2. Structural analysis of the Lap/GelMA hydrogel by FTIR confirmed the presence of Lap and GelMA in the hydrogel. The presence of a broader band in 3500 cm^−1^, 1630 and 1060 cm^−1^ was attributed to the structural and water O-H stretching, water deformation and Si-O stretching of the siloxane groups and to the deformation, respectively, characteristic of Lap. The FTIR spectrum of GelMA has an intense band in 1650 cm^−1^ related to the stretching of the band referring to amide I (–C=O). The band at 1500–1570 cm^−1^ corresponds to the deformation of C-N-H, while the band in the region between 3200 and 3400 cm^−1^ indicates the presence of peptide bonds, mainly the N-H stretch. The band in 3062 cm^−1^ was attributed to the stretch = C-H and in 1640 cm^−1^ was associated with the C=C stretching of GelMA groups which points to the interaction between gelatin and methacrylate anhydride. The Lap/GelMA hydrogel spectrum showed the characteristic bands of Lap and GelMA and agreed with previous work [27].

**Figure 3:**
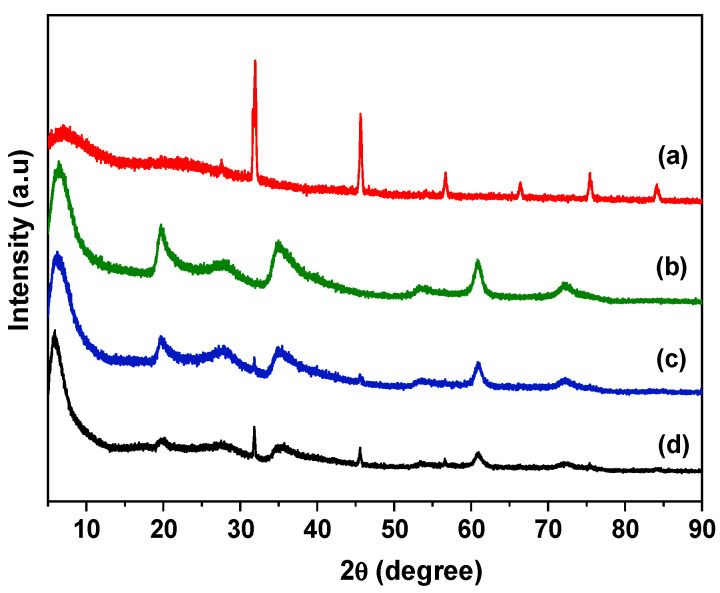
XRD patterns from (a) GelMA; (b) Lap; (c) hydrogel Lap/GelMA; (d) hydrogel GelMA/Lap (with Igarcure 2959).

The XRD patterns are shown in Figure 3. For GelMA (Figure 3 (a)), six main reflections were detected. The highest intensity reflection was observed at 2θ = 32° and also reflections at 46, 57, 67, 75 and 84° that were attributed to the polymer network [5,6,7,8]. For the Lap sample (Figure 3 (b)), the first reflection at 2θ = 6.5° (001 reflection) was associated with basal spacing of 1.36 nm, which is characteristic of Lap. Additional reflections were also observed at 20°, 29°, 35°, 55°, and 61° [18]. The reflection associated with 060 reflection occurred above 60° and it is indicative of a trioctahedral clay sample, characteristic of Lap. For the GelMA hydrogel (with or without iguarcure—Figure 3 (c,d)), the XRD patterns showed similarity with the Lap sample and the presence of two broad reflections of GelMA. The Lap/GelMA (Figure 3 (c,d)) hydrogels, 001 reflection associated with Lap phase shifted from 6.5° to 6.15°, corresponding to a basal spacing of 1.44 nm, indicating the presence of GelMA polymeric chains in the interlayer space of the clay mineral [18].

### 3.2. Thermogravimetric Analysis

**Figure 4:**
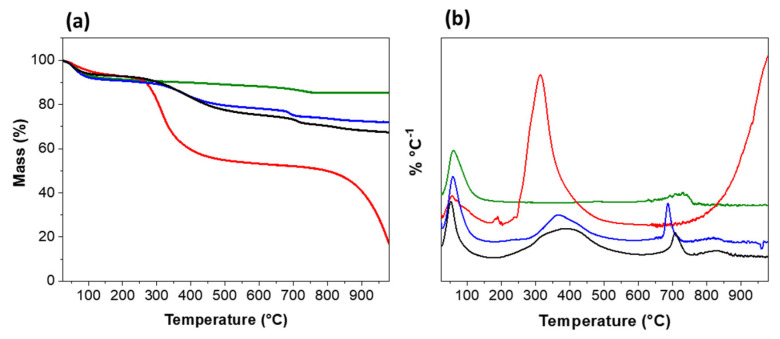
(**a**) TG/DTG and (**b**) derivative (DTG) curves of GelMA (red line); Lap (green line); hydrogel GelMA/Lap (blue line); hydrogel GelMA/Lap/Ig (black line).

The thermogravimetric (TG) curves are shown in Figure 4a, and the derivatives are in Figure 4b. The TG curves of the hydrogel, with and without a photoinitiator, and its precursors are shown in Figure 4a. Lap and GelMA exhibited distinct degradation patterns, with Lap (Figure 4a) showing more excellent thermal stability than GelMA. For nanoclay, two different mass loss events were observed, the first at 59 °C with a loss of 8%, related to water loss, and the second at ~727 °C with a mass loss of 2.8% related to dehydroxylation of the surface of the nanoclay (Figure 4b).

For GelMA (Figure 4b), two events were also observed: the first at 56 °C with a mass loss of 6.27% and the second at ~313 °C with a mass loss of 37.5%, which were attributed to the loss of surface groups, probably from the incorporated acrylate groups [28]. Above 700 °C, degradation of the organic polymeric structure occurred. Lap/GelMA (Figure 4b) presented four mass loss events at 59 °C with a mass loss of 8.5%; 368 °C with a mass loss of 11.1% and 688 °C and 815 °C with a mass loss of 3.1% and 1.8%, respectively. For Lap/GelMA/Igarcure (Figure 4b), four mass loss events were observed: 6.95% at 51 °C, 17.6% at 386 °C, 4.2% at 710 °C, and 2.5% at 833 °C. The addition of nanoclay improved the thermal stability of the hydrogel. The shift in peaks with increasing temperature observed in the DTG (Figure 4b) suggests that the polymer/clay interaction increased the chemical stability of the material, and the photoinitiator displaced the events verified in the hydrogel without its presence, indicating the effectiveness of its action (Figure 4b) [29]. The analysis confirmed the interaction between Lap and GelMA.

### 3.3. Rheology

**Figure 5:**
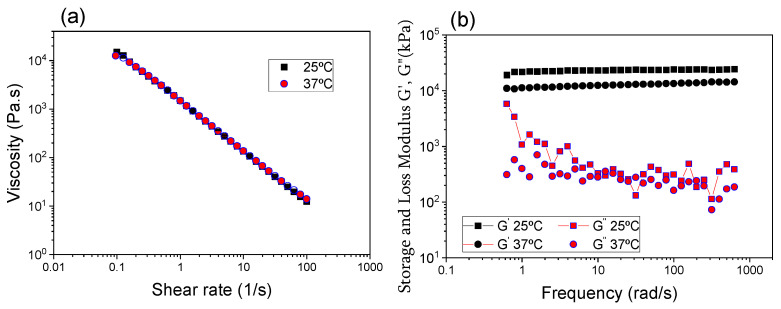
Rheological analysis of the Lap/GelMA hydrogel: (**a**) viscosity as a function of shear rate; (**b**) storage modules (G’) and loss module (G”).

The rheological properties of the Lap/GelMA hydrogel are shown in Figure 5. The viscosity as a function of shear rate is shown in Figure 5a and the loss and storage modulus as a function of oscillatory frequency is shown in Figure 5b. The Lap/GelMA hydrogel showed thixotropic behavior concerning its viscosity, which could decrease the viscosity with increasing application time at a given shear rate. This phenomenon is reversible and is a consequence of the gradual destruction of the structure composed of the particles of the dispersed phase, whose binding force does not resist the shear. When the shear force ceases, the system recovers its original viscosity [23], which is a fundamental characteristic for materials that will be used in an injectable way.

The modules of loss (G’) and storage (G”) of the Lap/GelMA hydrogel were monitored as a function of the oscillatory frequency. G’ and G” were studied to evaluate the viscoelasticity of the material. The deformation suffered as a function of the applied stress described an intermediate behavior (with a delay angle, δ, between 0° and 90°) between an elastic material (Hookian) and an ideal viscous material (Newtonian). This classifies the material as viscoelastic with a greater elasticity component, since G’ > G”, being in agreement with the work of Sheikhi et al., in which the authors, evaluating the viscoelasticity of laponite hydrogels with gelatin at different concentrations and media, observed that the hydrogel prepared in PBS or DMEM presented a higher viscoelastic modulus (G’ > G”) than the gel prepared in Milli Q water, possibly due to the formation of aggregates with rapid phase separation under stress, which increases the risk of complications during hydrogel injection. They further observed that if G’ ˂ G”, the viscoelasticity greatly decreases as a result of solid precipitation. The best results were found for the hydrogel prepared in Milli Q water, as a more homogeneous hydrogel was formed [23]. According to Li et al., 2016 [30], variations in the rheological properties of a composite with Lap and a polymer can occur when the Lap structures are partially exfoliated, intercalated or aggregated within the polymer matrix. The G’ > G’’ can be attributed to the strong interaction or increased cross-linking between polymer chains and clay platelets. The G’ value of the Lap/GelMA composite did not decrease over a wide frequency range, which may indicate the absence of excess non-intercalated aggregates that could impair the hydrogel injectability.

### 3.4. Morphology

**Figure 6:**
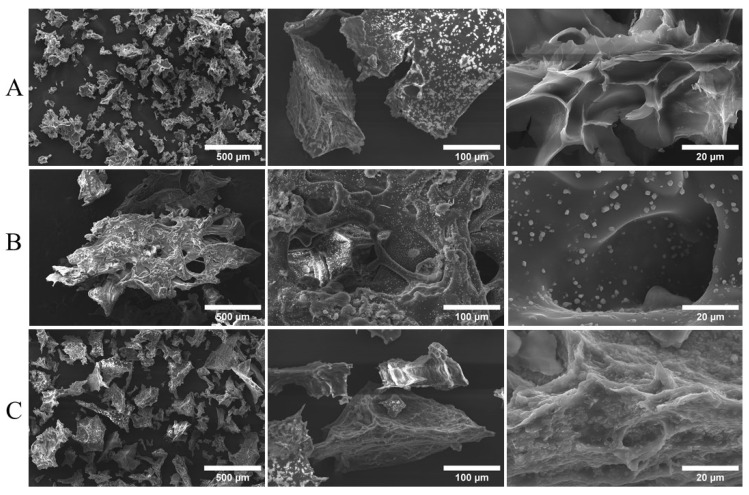
SEM images for (**A**) Lap 6%; (**B**) GelMA 1%; (**C**) Hydrogel Lap/GelMA.

SEM images of the Lap/GelMA composite and precursors are shown in Figure 6. The SEM image of Lap (Figure 6A) consisted of particles of different sizes on a micrometer scale and with a rough and irregular surface, which agrees with a previous study [14,15,16,17,18,19,20,21,22,23,24,25,26,27,28,29,30,31]. The image of the GelMA hydrogel is shown in Figure 6B, showing an interconnected pore structure. The degree of substitution of methacryloyl in gelatin influences the size of the pores in the hydrogel. High substitution degrees tend to reduce the pore size [10]. For the Lap/GelMA composite (Figure 6C), the SEM image suggests a more compact and less irregular surface, although still porous. The presence of pores is desirable since they facilitate cell adhesion, proliferation, and nutrition—essential characteristics for scaffolds to be used in tissue engineering [17,18,19,20,21,22,23,24,25,26,27,28,29,30,31,32].

### 3.5. Assay Toxicity

This test is based on the premise that bioactive compounds are majorly toxic when used at high doses. In vivo lethality in a simple organism can be used as a screening parameter in discovering new bioactive materials. Previous work suggests a good correlation between Artemia salina assay activity and cytotoxicity against some tumor cell lines [33]. Nguta et al., 2011 [33] observed that the increase in Artemia salina mortality was proportional to the increase in sample concentration, which provided linearity in the dose/effect relationship and determination of the mean lethal dose*—*LD 50 [25,26,27,28,29,30,31,32,33].

**Figure 7:**
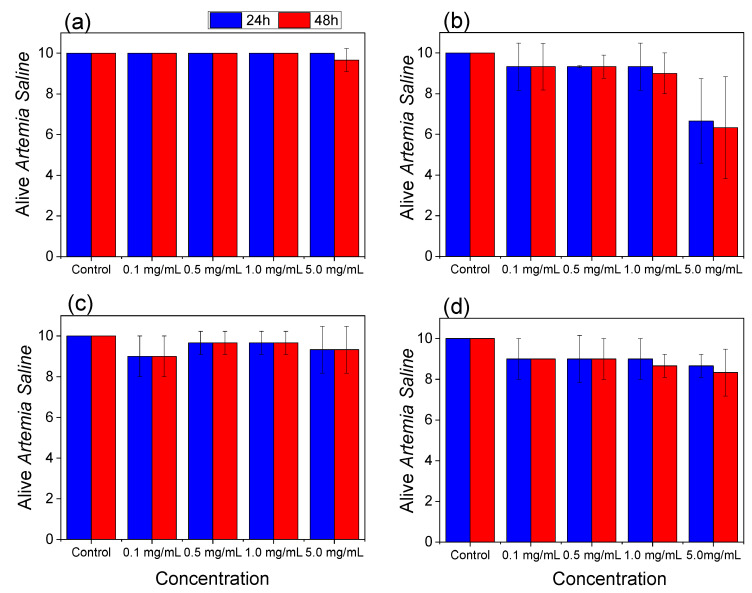
*Artemia salina* lethality assay—(**a**) Lap; (**b**) GelMA; (**c**) Lap/GelMA hydrogel; (**d**) Lap/GelMA hydrogel (with igarcure). The control group were compared to the other groups, with a statistically significant difference for *p* ≤ 0.05.

The *Artemia salina* assay was performed for the hydrogel, precursors, and the photoinitiator used in synthesis, Igarcure 2959 (Ig) (Sigma), at concentrations of 5 mg mL^−1^; 1 mg mL^−1^; 0.5 mg mL^−1^ and 0.1 mg mL^−1^. Figure 7 shows that the photoinitiator Igarcure 2959 (Ig) did not show toxicity. Furthermore, analyzing the precursors alone, laponite did not show toxicity at any concentrations. In contrast, the GelMA sample only at the highest concentration (5 mg mL^−1^) showed slight toxicity but above the DL 50. However, the combination of the two precursors in developing the Lap/GelMA hydrogel showed no toxicity at all concentrations evaluated, thus indicating safety for use at the concentrations tested. Compared with the control group, where only saline water was tested, the other groups in this study did not show a statistically significant difference (*p* ≤ 0.05) at 24 and 48 h, thus demonstrating the non-toxicity of the materials—demonstrating a percent survival with about 90.0% in all groups.

### 3.6. In Vitro Cytotoxicity

**Figure 8:**
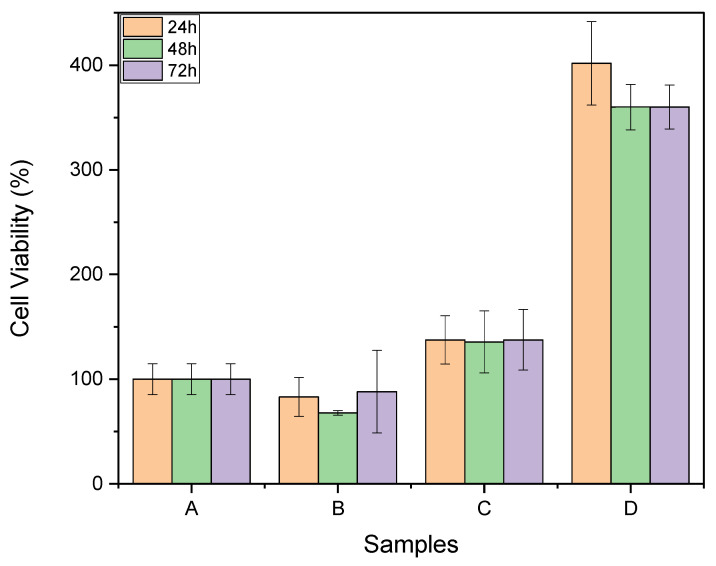
Cell viability tests by MTT at different times (24, 48 and 72 h) for samples: (A) Control; (B) GelMA; (C) Lap; (D) Hydrogel Lap/GelMA (with Iguarcure). The control groups were compared to the other groups, with a statistically significant difference for *p* ≤ 0.05.

The evaluation of the biocompatibility of the Lap/GelMA hydrogel, the precursors, and toxicity against mouse bone marrow stem cells was carried out using an MTT assay. The experiments were conducted in three periods: 24, 48, and 72 h. No significant toxicity was observed compared to the control (positive control), i.e., a cell culture well with no material for either the hydrogel or the precursors. The results (Figure 8) of the analysis of cell viability using the MTT test demonstrated no cytotoxicity in sample testing compared with a group control in 24, 48, and 42 h. The groups Laponite 6% and GelMA 1% did not present results more statistically significant than the control group. The group with Laponite + GelMA in its composition presented higher cell viability than the control group and GelMA 1% and Laponite 6% samples. Orafa et al. evaluated the biocompatibility of nanofibrous PLA scaffolds functionalized with Laponite/amoxicillin through an MTT assay. They found that the cells remained viable after contact with the tested material. Kulkarni et al. tested the biocompatibility of a GelMA hydrogel through an MTT assay, and the cell viability found was above 80% [34,35].

### 3.7. Osteogenic Differentiation

**Figure 9:**
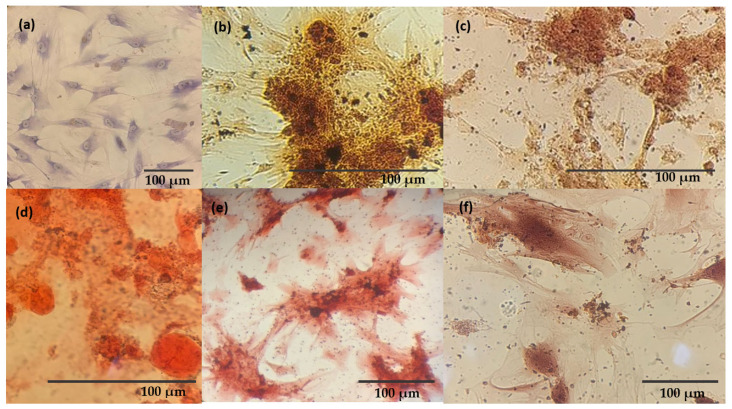
Osteogenic differentiation assessment by Alizarin Red: (**a**) undifferentiated stem cells; (**b**) Osteoblasts after 21 days of treatment in contact with the hydrogel Lap and stained with Alizarin Red; (**c**) Osteoblasts after 21 days of treatment in specific media and contact with the hydrogel Lap and stained with Alizarin Red; (**d**) Osteoblasts after 21 days of treatment in specific media and contact with the hydrogel GelMA and stained with Alizarin Red; (**e**) Osteoblasts after 21 days of treatment in contact with the hydrogel Lap/GelMA and stained with Alizarin Red; (**f**) Osteoblasts after 21 days of treatment in specific media and contact with the hydrogel Lap/GelMA and stained with Alizarin Red.

The evaluation of the differentiation potential of mouse bone marrow stem cells in contact with the Lap/GelMA hydrogel and its precursors through staining with Alizarin Red after 21 days of treatment with and without the specific inducing medium confirmed the induction potential of differentiation of undifferentiated stem cells to osteoblasts (Figure 9).

Calcium phosphate deposits released from osteoblasts covered the cell surface, appearing as red nodules after staining with Alizarin Red. This behavior occurred in the Laponite-only and Lap/GelMA hydrogel groups. When the materials were in contact with stem cells with and without adding a specific inducing medium, they showed the same differentiation pattern in the presence and absence of the inducing medium. This leads to the conclusion that the material has the potential for inducing cell differentiation in osteoprogenitor cells even in the absence of a specific inducing medium (Figure 9b,c,e,f). However, when only GelMA was placed in contact with the cells, with and without a specific inducing medium, was cell differentiation observed only in the group where the inducing medium was added (Figure 9d). Figure 9a shows the control group, in which stem cells that are still undifferentiated are observed, since neither the inducing medium for osteogenic differentiation was added nor the materials for testing.

This result can be attributed to the presence of laponite because the clay mineral can induce cell differentiation even in the absence of any other inducing factor. The mechanisms related to osteogenic differentiation promoted by clays remain little known. However, the osteogenic effects of their degradation products are always cited. The laponite, Si(OH)_4_, Mg^2+^, and Li^+^ have improved osteogenic cell function since orthosilicic acid promotes type I collagen synthesis and differentiation into osteoblasts. Magnesium ions are involved in the activation of regulatory pathways of osteogenesis HIF-1α (hypoxia-induced factor-1 alpha) and PGC-1α (Peroxisome proliferator-activated receptor-gamma coactivator (PGC)-1alpha) and are essential for the adhesion of integrin to the surface of biomaterials, and lithium activates the response of canonical Wnt-responsive osteogenic genes through the inhibition of GSK3β (Glycogen synthase kinase-3 beta) [12].

## 4. Conclusions

The acute toxicity, biocompatibility, and cell differentiation ability of the Lap/GelMA hydrogel were investigated. FTIR and XRD suggested the formation of polymer intercalation hybrids in the clay matrix. TG/DTG analyses confirm the interaction and displacement of events after insertion of the photoinitiator, indicating more significant interaction. The formation of a porous hydrogel matrix was confirmed using SEM imaging, which is a crucial requirement for cell adhesion, proliferation, and nutrition. The initial results of the rheological study indicated that the sample has a viscosity that is adequate for injectability. The Lap/GelMA hydrogel did not show toxicity in the *Artemia salina* assay at the tested concentrations. The evaluation of biocompatibility by the MTT assay showed cell viability more significant than 80% for the Lap/GelMA hydrogel, which infers the absence of toxic effects on cells and good cell growth after 72 h. The evaluation of the cell differentiation capacity of the hydrogel showed, through Alizarin Red staining, the cell differentiation of stem cells into osteogenic cells when the two precursors were united in the hydrogel. The absence of toxicity and cytotoxicity observed in in vitro tests enables the material to be tested in vivo. The results show that the Lap/GelMA hydrogel can be a viable strategy for application in bone tissue engineering as an osteoinductive material. However, future investigations must be carried out to optimize the material, mainly with regard to the biological characteristics and biocompatibility with specific cell types that can enable it, definitively, to be used in bone tissue engineering. The material’s injectability is another point that raises the need for more specific studies since the possibility of injecting it into bone defects with undefined geometry is a highly desired feature in tissue engineering.

## Data Availability

Not applicable.

## Supplementary Materials

The following supporting information can be downloaded at: https://www.mdpi.com/article/10.3390/jfb14020074/s1, Figure S1: Images of cell viability tests by MTT in differents time points.
