# Supplementary material for: Hydrogel Based on Nanoclay and Gelatin Methacrylate Polymeric Matrix as a Potential Osteogenic Application
Source: J Funct Biomater. 2023 Jan 29;14(2):74. doi: 10.3390/jfb14020074 (PMC9961749; doi:10.3390/jfb14020074)
Supplement: Supplementary file 1 [file jfb-14-00074-s001.zip › jfb-2082997-supplementary.pdf]

Supplementary Materials:

Hydrogel based on nanoclay and gelatin methacrylate polymeric matrix as a potential osteogenic application

Danielle B. Andrade[1], Leticya L. S. Soares[2], Francisca L. A. Cardoso[2], Idglan S. Lima[1], Jhaemely G. V. Silva[1], Maria A. M. Carvalho[2], Maria G. Fonseca[3], Guilherme de C. Brito, Francisco Eroni P. Santos[1], Josy A. Osajima[1,4], Anderson O. Lobo[1*] and Edson C. Silva-Filho[1,4*]

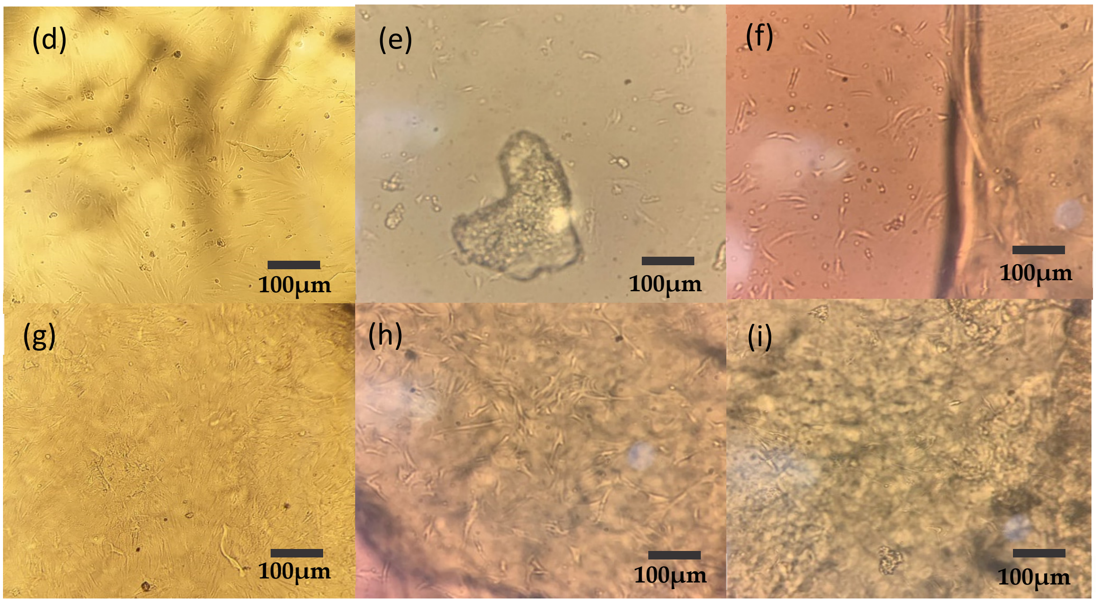

Figure S1. Images of cell viability tests by MTT : (d) GelMA 48 h, (e) Lap 48h, (f) Lap/GelMA 48 h; (g)GelMA 72 h, (h)Lap 72h, (i) Lap/GelMA 72 h.
